# The complete mitochondrial genomes of the freshwater mussel *Ortmanniana ligamentina* (Lamarck, 1819): male and female mitotypes

**DOI:** 10.1080/23802359.2025.2500528

**Published:** 2025-05-12

**Authors:** Katy Klymus, Jason Coombs, Dannise Ruiz-Ramos, Aaron Maloy, Christopher Barnhart

**Affiliations:** ^a^ U.S. Geological Survey, Columbia Environmental Research Center, Columbia, MO, USA; ^b^ Northeast Fishery Center, U.S. Fish and Wildlife Service, Lamar, PA, USA; ^c^ Department of Natural Sciences, University of Maryland Eastern Shore, Princess Anne, MD, USA; ^d^ Department of Biology, Missouri State University, Springfield, MO, USA

**Keywords:** Unionida, species conservation, double uniparental inheritance

## Abstract

Freshwater mussels of the Unionida order are important to freshwater ecosystems but are highly imperiled worldwide. Improving our understanding of these species is crucial to their continued conservation. Some Unionid mussels exhibit double uniparental inheritance (DUI) in which individuals have two mitochondrial genomes. Of those species with DUI, sequences of the female mitotype are most prevalent in genetic databases. Here, we demonstrate the ability to recover both mitotypes of Ortmanniana ligamentina (Lamarck, 1819) from a non-lethal collection method coupled with high-throughput sequencing. Increased male mitotype sequence representation facilitates understanding Unionid genetic diversity and development of molecular tools for species detection.

## Introduction

A unique feature in some bivalves is the double uniparental inheritance (DUI) of mitochondrial DNA (Fisher and Skibinski 1990; Skibinski et al. 1994; Zouros et al. 1994; Walker et al. 2006). In this system, individuals have two separate mitochondrial genomes, or mitotypes, that are passed onto offspring. This differs from the more common mode of metazoan mitochondrial inheritance (strict maternal inheritance [SMI]) in which only the maternal mitochondrial genome is passed to offspring (Breton et al. 2007; Passamonti et al. 2011). Within bivalves with DUI, female mussels are believed to be homoplasmic, having the female mitotype in their somatic and gametic tissues, whereas male mussels are heteroplasmic (Garrido-Ramos et al. 1998; Soroka 2020). Male individuals’ somatic tissue contains the female mitotype, but their gametic tissue predominantly contains the male mitotype. Sequence divergence between these mitotypes is large, with differences of up to 52% between female and male mitotypes of the same species (Doucet-Beaupré et al. 2010; Soroka 2020). Among the bivalve taxa exhibiting DUI are species from at least three Unionida families (Unionidae, Margaritiferidae, and Hyriidae) (Soroka and Burzyński 2018).

Unionida are among the most threatened freshwater taxa (Lydeard et al. 2004; Galbraith et al. 2015; Graf and Cummings 2021; USFWS 2024). New tools for species monitoring, such as environmental DNA could be useful for their conservation (Prié et al. 2021; Klymus et al. 2021). Development of eDNA assays requires extensive genetic sequence data but currently male mitotype sequence data is limited in public databases. Here we assemble both female and male mitotypes of the Mucket, *Ortmanniana ligamentina* (Lamarck, 1819) as part of an effort to increase male mitotype representation in genetic databases.

## Materials and methods

### Sample collection and extraction

**Figure 1.**
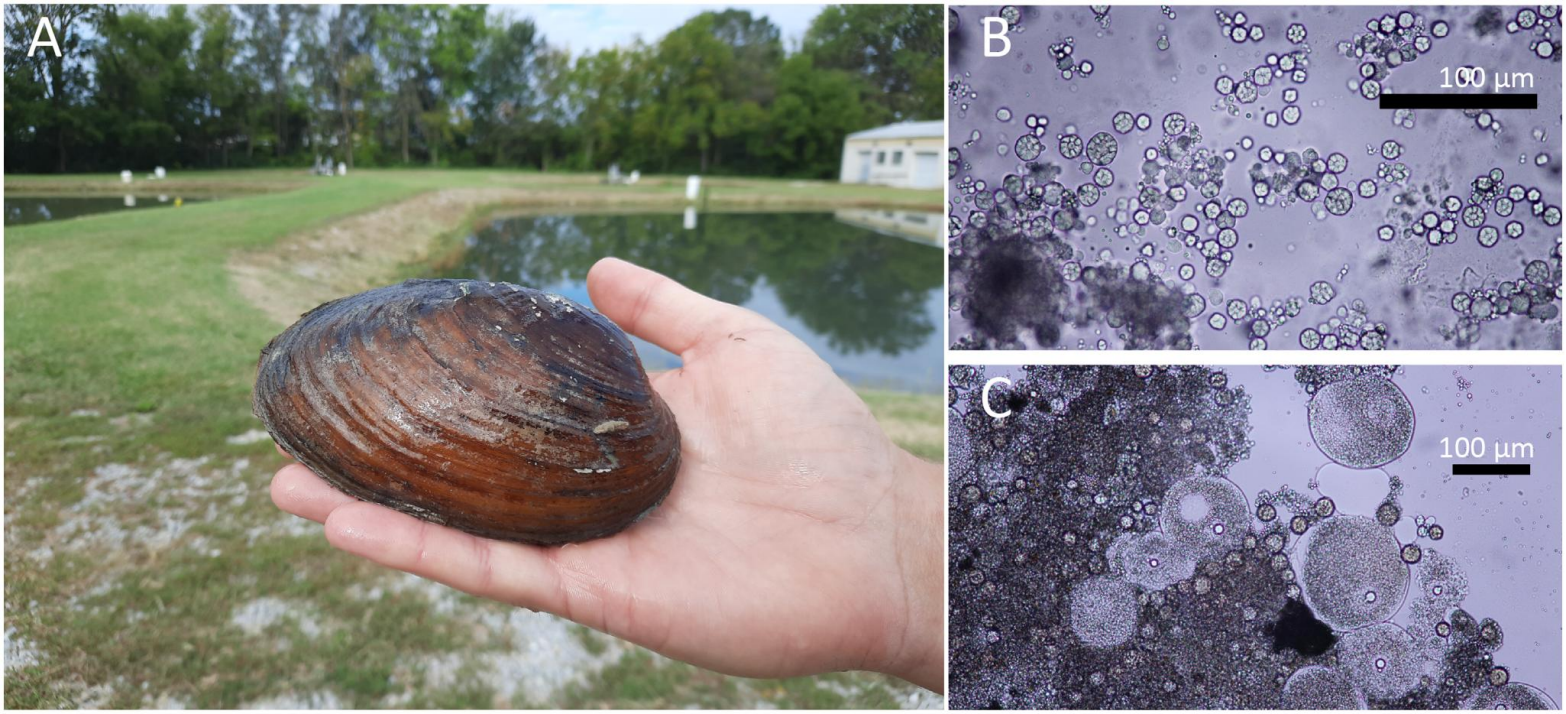
(A) Mucket mussel. Photo taken by Katy Klymus at Columbia Environmental Research Center, Missouri, USA. (B) Magnified image (40X) of male gametic fluid removed *via* a syringe. The material was used to verify the sex of individual mussels and for sequencing. Sequencing of the male gametic tissue ensured we recovered the male mitotype genome. We were also able to recover the female mitotype genome from the same sample. Photo taken by Chris Barnhart at Missouri State University. (C) Magnified image (20X) of female gametic fluid removed *via* a syringe. The material was used to verify the sex of individual mussels. We did not sequence female gametic tissue. Photo taken by Chris Barnhart at Missouri State University.

Individuals of *Ortmanniana ligamentina* (Figure 1(A)) were collected by the Missouri Department of Conservation (Saint Francis River, Missouri; 37° 10′ N; 90° 28′ W) 1 June 2020 and the Pennsylvania Fish and Boat Commission (Allegheny River, Pennsylvania; 41° 28′ N; 79° 29′ W) 24 May 2016. Each specimen was identified by shell morphology in the field and confirmed by later examination of the mantle and gill morphology by at least two workers familiar with the local range of variation and similar species (McMurray, Faiman, et al. 2012; Pennsylvania Fish & Boat Commission Division of Environmental Services 2018). Tissue samples consisting of 100 µL of gonadal fluid from Missouri mussels were obtained with a 1-mL syringe and 20–18 g needle. A hypodermic needle inserted into the anterior visceral mass was slowly rotated as the syringe withdrew fluid containing mainly male gametes and precursor cells. With care, samples can be taken without causing mortality (Shiver, 2002; Tsakiris et al. 2016). Microscopy of the gonad fluid was used to identify the sex of the individual mussels from Missouri (Figure 1(B,C)) and a sample from an identified male individual was used in subsequent sequencing. Sample of a gill biopsy collected from a female Pennsylvania specimen was also sequenced to validate the data collected from the syringe method. Samples were preserved in 95% EtOH. Total genomic DNA was extracted with either the IBI Scientific gMAX Mini Genomic DNA kit or Qiagen DNeasy Blood and Tissue kit. Extracted DNA and tissue samples (B065) from Missouri are archived at the Columbia Environmental Research Center, Columbia, Missouri, USA (https://www.usgs.gov/centers/columbia-environmental-research-center, Dr. Catherine Richter, crichter@usgs.gov). Extracted DNA (B16-023) from the Pennsylvania specimen is archived at the Northeast Fishery Center, Lamar, Pennsylvania, USA Center (https://www.fws.gov/office/northeast-fishery-center).

### Genomic sequencing, assembly, and annotation

**Figure 2.**
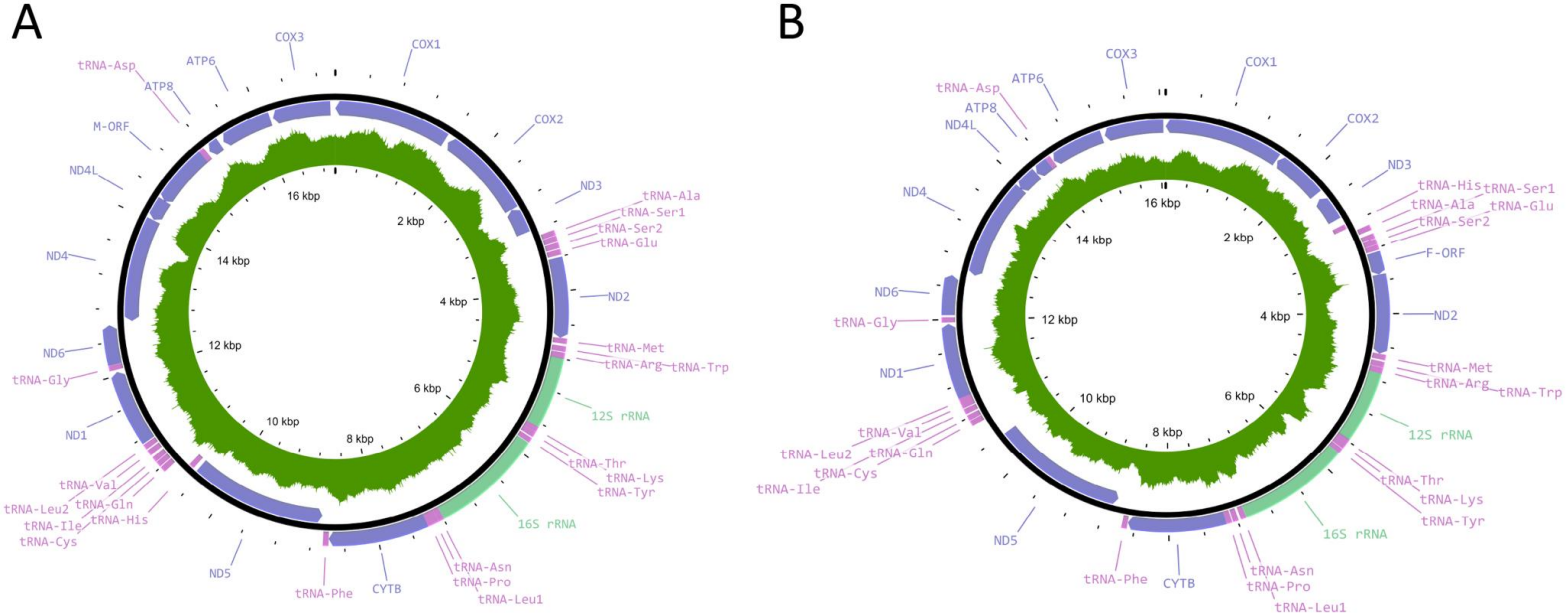
Mitogenome maps of (A) the male mitotype (accession number PP103562) from the Missouri individual (B065) and (B) the female mitotype (accession number PP103563) from the Missouri individual (B065). The map of the female mitotype from the Pennsylvania individual B16-023 (accession number PQ064524) is the same as that of the female mitotype from Missouri. The outer track shows the gene features and positions, with purple representing the coding DNA sequence, pink representing tRNAs, and light green representing rRNAs. The dark green inner track represents read depth for each assembly.

Library preparation and sequencing took place at either the University of Missouri Genomics Technology Core or the Northeast Fishery Center. Libraries were prepared from genomic DNA per manufacturer’s protocol with reagents supplied in the Illumina DNA Prep or Nextera XT preparation Kits. Final libraries were bead purified and sequenced on a NovaSeq 6000 platform (Illumina, San Diego, CA) with a 2 × 150 or 2 × 250 bp paired-end read kit. Adapters and low-quality bases were removed before merging paired-end reads. Merged reads were *de novo* assembled using the Tadpole assembler in Geneious to obtain a draft list of contigs. From the Missouri specimen, a total of 502,682,066 raw sequences were recovered resulting in 145,843,052 merged reads. From the Pennsylvania specimen, a total of 15,449,292 reads were recovered, resulting in 6,346,335 merged reads. Gene annotations from *Lampsilis siliquoidea* (GenBank accession MF326973) were temporarily mapped based on similarity to a subset of contigs which fell within the expected mitogenome size range. Temporary annotations were used to identify contigs of mitochondrial origin. Sequences of mitochondrial origin served as the seed sequence against which all merged reads were mapped over successive iterations. Reads successfully mapped were then *de novo* assembled to recover two circular mitochondrial genomes. Gene locations were determined based on similarity to other Unionid species and verified using MitoAnnotator (Iwasaki et al. 2013). Gene arrangement and presence of the male and female open reading frames were used to identify the longer mitogenome as the male mitotype. All trimming and assembly steps were conducted using Geneious Prime version 2022.0.2 (Biomatters Ltd., Auckland, New Zealand). The average read depth of the mitogenomes from the Missouri specimen was 60 while that of the Pennsylvania specimen was 146. Genome maps were made using Proksee (Grant et al. 2023) (Figure 2(A,B), Supplemental Figures S1 and S2).

The new genomes were relationally assessed through generation of phylogenetic trees using sequence data from GenBank of 36 Unionida species with complete male and female mitogenome data (Table 1). Genes were extracted and aligned within Geneious, and concatenated and exported in SequenceMatrix version 1.9 (Vaidya et al. 2011). Trees were generated with the FastTree (Price et al. 2009, 2010) plugin in Geneious using default settings and rooted with *Pseudunio marocanus* a species from the subfamily Margaritiferinae. Tree visualization was with Interactive Tree of Life (iTOL) (Letunic and Bork 2021).

**Table 1.** A List of NCBI accession numbers and associated publications for mitogenomes of 36 unionid mussel species and 37 sequences used for phylogenetic tree construction.

Subfamily	Species	Female accession	Male accession
Unioninae	*Anodonta anatina*	KF030964 (Soroka and Burzyński 2015)	KF030962 (Soroka and Burzyński 2016)
Gonideinae	*Chamberlainia hainesiana*	MK994770 (Froufe et al. 2020)	MK994771 (Froufe et al. 2020)
Unioninae	*Cuneopsis celtiformis*	MW464617	MZ571520 (Wu et al. 2022)
Rectidentinae	*Hyriopsis bialata*	MW242816 (Zieritz et al. 2021)	MW242817 (Zieritz et al. 2021)
Gonideinae	*Lamprotula caveata*	KX060991	KX091842
Gonideinae	*Lamprotula gottschei*	KJ018924 (He et al. 2016)	KJ627225
Unioninae	Lamprotula tortuosa* *(Aculamprotula tortuosa)*	KC109779 (Wang et al. 2013)	KC441487
Unioninae	*Lanceolaria lanceolata*	KJ144818 (Wang et al. 2016)	KJ775864
Ambleminae	*Lampsilis powellii*	MF326971 (Robicheau et al. 2018)	MF326972 (Robicheau et al. 2018)
Ambleminae	*Lampsilis siliquoidea*	MF326973 (Robicheau et al. 2018)	MF326974 (Robicheau et al. 2018)
Rectidentinae	*Lens contradens*	MW242812 (Zieritz et al. 2021)	MW242813 (Zieritz et al. 2021)
Gonideinae	*Microcondylaea bonellii*	MK994772 (Froufe et al. 2020)	MK994773 (Froufe et al. 2020)
Subfamily	Species	Female accession	Male accession
Pseudodontinae	Monodontina vondembuschiana* (*Pseudodon vondembuschianus)*	MK994774 (Froufe et al. 2020)	MK994775 (Froufe et al. 2020)
Unioninae	*Nodularia douglasiae*	MT447062	KP970613
Ambleminae	*Ortmanniana ligamentina MO*	PP103563 (this study)	PP103562 (this study)
Ambleminae	*Ortmanniana ligamentina PA*	PQ064524 (this study)	N/A
Gonideinae	*Parvasolenaia rivularis*	KX966393	KY007142
Rectidentinae	*Physunio superbus*	MW242814 (Zieritz et al. 2021)	MW242815 (Zieritz et al. 2021)
Pseudodontinae	*Pilsbryoconcha exilis*	MK994776 (Froufe et al. 2020)	MK994777 (Froufe et al. 2020)
Ambleminae	*Potamilus alatus*	KU559011 (Wen et al. 2017)	KU559010 (Wen et al. 2017)
Gonideinae	*Potomida littoralis*	KT247374 (Froufe et al. 2016)	KT247375 (Froufe et al. 2016)
Unioninae	*Pseudocuneopsis sichuanensis*	MZ571510 (Wu et al. 2022)	MZ571518 (Wu et al. 2022)
Margaritiferinae	*Pseudunio marocanus*	KY131953 (Lopes-Lima et al. 2017)	KY131954 (Lopes-Lima et al. 2017)
Unioninae	*Pyganodon grandis*	FJ809754 (Breton et al. 2009)	FJ809755 (Breton et al. 2009)
Subfamily	Species	Female accession	Male accession
Ambleminae	*Quadrula quadrula*	FJ809750 (Breton et al. 2009)	FJ809751 (Breton et al. 2009)
Rectidentinae	*Rectidens sumatrensis*	MW242818 (Zieritz et al. 2021)	MW242819 (Zieritz et al. 2021)
Unioninae	*Sinanodonta woodiana*	KM272949 (Zhang et al. 2016)	MH349356 (Burzyński and Soroka 2018)
Gonideinae	*Sinohyriopsis cumingii*	FJ529186 (Huang et al. 2013)	KC150028
Gonideinae	Solenaia carinata* *(Sinosolenaia carinata)*	KC848654 (Huang et al. 2013)	KC848655 (Huang et al. 2013)
Gonideinae	Solenaia oleivora* *(Sinosolenaia oleivora)*	KF296320 (Huang et al. 2015)	KY007143
Unioninae	*Tchangsinaia piscicula*	KP273584	MZ571519 (Wu et al. 2022)
Unioninae	*Unio crassus*	KY290446 (Burzyński et al. 2017)	KY290448 (Burzyński et al. 2017)
Rectidentinae	Unio delphinus* *(Hyriopsis bialata)*	KT326917 (Fonseca et al. 2017)	KT326918 (Fonseca et al. 2017)
Unioninae	*Unio pictorum*	HM014130 (Soroka and Burzyński 2010)	MH349357 (Burzyński and Soroka 2018)
Unioninae	*Unio tumidus*	KY021076 (Soroka and Burzyński 2018)	KY021073 (Soroka and Burzyński 2018)
Unioninae	*Utterbackia peninsularis*	HM856636 (Breton et al. 2011)	HM856635 (Breton et al. 2011)
Unioninae	*Venustaconcha ellipsiformis*	FJ809753 (Breton et al. 2009)	FJ809752 (Breton et al. 2)

Accession numbers without references are unpublished. For the female *Ortmanniana ligamentina*, the MO and PA suffixes refer to the location of capture, Missouri and Pennsylvania, respectively. An asterisk (*) indicates a name change according to the MolluscaBase (MolluscaBase 2025) database with the first name being the name recorded in GenBank and the second name being the recently changed name.

## Results

Sampling of gonadal fluid resulted in genomic DNA extract containing 0.12% mitochondrial reads for the male mitotype and 0.0034% mitochondrial reads of the female mitotype. This discrepancy is expected, as less somatic tissue containing the female mitotype was present in the collected gonadal fluid.

**Figure 3.**
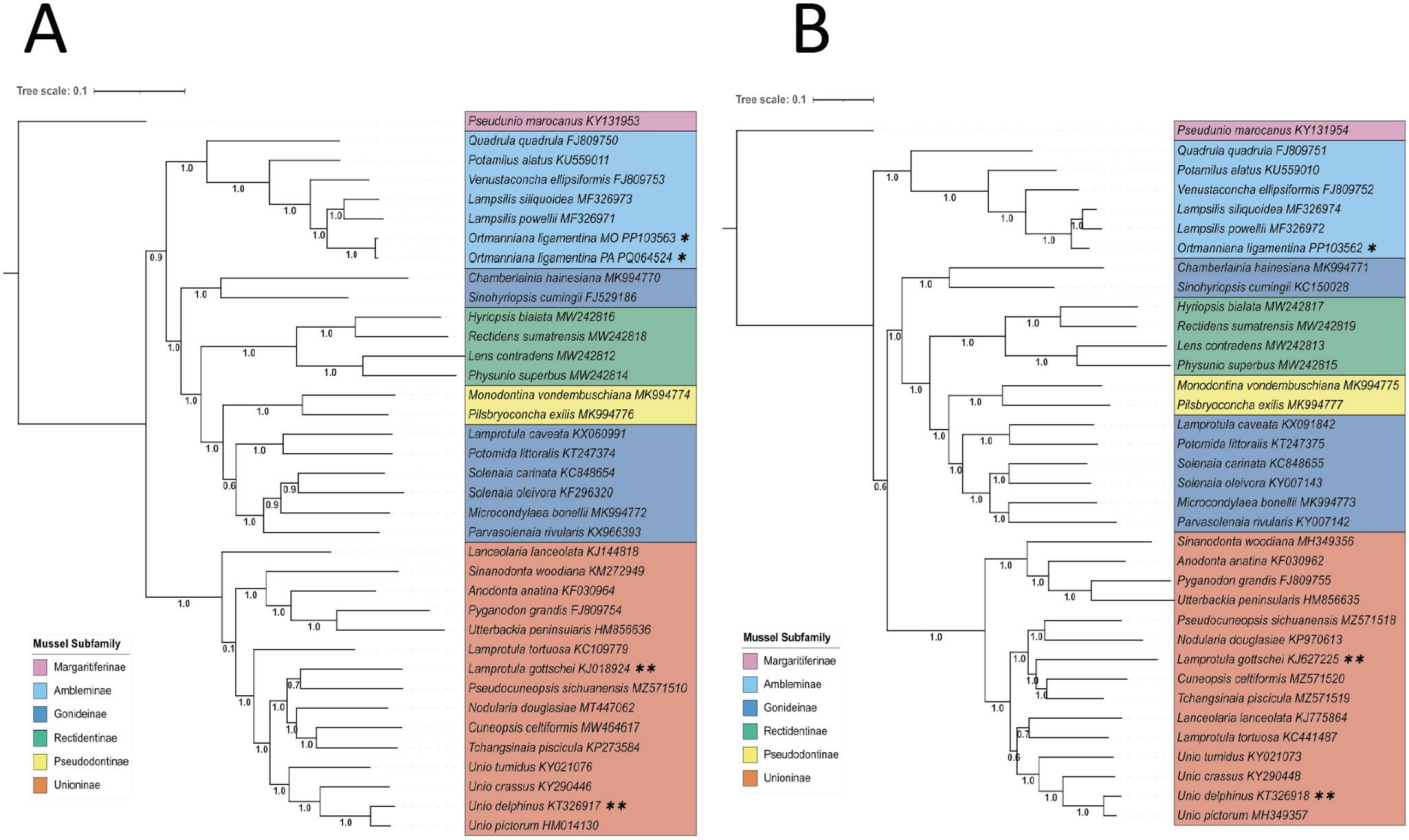
Inferred phylogenetic trees based on the 13 coding genes and two ribosomal RNAs of the mitochondrial genome using approximately maximum likelihood methods through FastTree (Price et al. 2009, 2010). Numbers denote Shimodaira–Hasegawa test support values of the associated split. An asterisk (*) indicates the individuals genotyped in this study. Subfamily names are indicated to the right of each clade. Double asterisks (**) indicate two individuals with different subfamily designations than that in which they group with. The sequences and their accession numbers used can be found in Table 1. (A) Phylogenetic tree of the female mitotypes. (B) Phylogenetic tree of male mitotypes.

From the Missouri specimen, the assembled male mitogenome (GenBank accession PP103562) was 17,015 bp, and the female mitogenome (GenBank accession PP103563) was 16,070 bp (Figure 2(A,B), Supplementary Figures S1 and S2). The length of the *COX2* region heavily influenced the difference in length; the female *COX2* region was 681 bp, while the male *COX2* was 1242 bp. The female mitogenome of the Pennsylvania specimen (GenBank accession PQ064524) was 16,075 bp in length. Phylogenetic analyses place the two female mitotype genomes as sister to one another within the Ambleminae subfamily and sister to *Lampsilis siliquoidea* and *Lampsilis powellii* (Figure 3(A)). Similarly, the sequenced male mitotype genome was placed among male mitotype genomes of other Ambleminae individuals (Figure 3(B)). The trees of male and female mitotypes have similar topology, with sequences grouping in the same subfamily clades. Interestingly, the Unioninae clade changes places with the Ambleminae clade between the two mitotype topologies, but this could be an artifact of limited sampling.

Although not a focus of this study, the use of sequences from GenBank reveals discrepancies between current taxonomy using MolluscaBase (MolluscaBase 2025) and the species identification of the downloaded sequences for two individuals (*Unio delphinus* (KT326917/KT32918) and *Lamprotula gottschei* (KJ018924/KJ627225) (Figure 3(A,B), Table 1). These results suggest that these specimens might have been mis-identified in the original study or that further systematic study of these species is warranted.

## Discussion and conclusions

We report the first complete male mitotype genome of *O. ligamentina* (GenBank accession PP103562) and 2 complete female mitotype genomes (GenBank accession PP103563 and PQ064524). Our study’s phylogenetic placement of all three mitogenomes is supported by the current literature in that *O. ligamentina* is in the Ambleminae subfamily and sister to *Lampsilis siliquoidea* and *Lampsilis powellii* (Inoue et al. 2020; Stodola et al. 2021). We also demonstrate the ability to sequence and recover both mitotypes from non-lethal collection methods, using high throughput sequencing of gonadal tissue taken with a syringe. Previously, Sanger sequencing of the different mitotypes required sacrificing the animal to dissect clean gonadal tissue to avoid female mitotype contamination (Breton et al. 2006). This non-lethal method can increase the number of published male mitotype genomes which in turn can aid the study of DUI evolution and conservation practice. For instance, male mitotype sequences can be used to design molecular assays that target the identification of mussel male gametes (Prié et al. 2021). This application could identify reproductive triggers and spawning events which will be useful in the captive propagation being conducted to reintroduce populations in the wild.

## Supplementary Material

SuppFig1_031025.docx

SuppFig2_031025.docx

## Data Availability

Both raw read and final genome sequence data along with sample metadata are available in GenBank of the National Center for Biotechnology Information (NCBI) website (https://www.ncbi.nlm.nih.gov/) under the accession no. PP103562-3 and PQ064524. Other associate data include the BioProject (PRJNA633136), SRA (SRR27608898 for the two mitotypes obtained from specimen B065 and SRR31437301 for the mitotype obtained from specimen B16-023), and Bio-Sample numbers (SAMN39271813, SAMN42123394). The data are also available through USGS ScienceBase data release by Klymus et al. (2024).
