# Supplementary material for: The complete mitochondrial genomes of the freshwater mussel Ortmanniana ligamentina (Lamarck, 1819): male and female mitotypes
Source: Mitochondrial DNA B Resour. 2025 May 12;10(6):430–6. doi: 10.1080/23802359.2025.2500528 (PMC12077482; doi:10.1080/23802359.2025.2500528)
Supplement: SuppFig1_031025.docx [file TMDN_A_2500528_SM5903.docx]

**Supplementary Material**

The complete mitochondrial genomes of the freshwater mussel *Ortmanniana ligamentina* (Lamarck, 1819): male and female mitotypes

Katy Klymus^1*^, Jason Coombs^2^, Dannise Ruiz-Ramos^3^, Aaron Maloy^2^, Christopher Barnhart^4^

^1^U.S. Geological Survey, Columbia Environmental Research Center, 4200 New Haven Rd., Columbia, Missouri, USA

^2^U.S. Fish and Wildlife Service, Northeast Fishery Center, 308 Washington Ave., Lamar, Pennsylvania, USA

^3^University of Maryland Eastern Shore, 1 College Backbone Road, Princess Anne, Maryland, USA

^4^Missouri State University, 901 S. National Ave., Springfield, Missouri, USA

*Corresponding Author: Katy E. Klymus; [kklymus@usgs.gov](mailto:kklymus@usgs.gov); U.S. Geological Survey, Columbia Environmental Research Center, 4200 New Haven Rd., Columbia, Missouri, USA


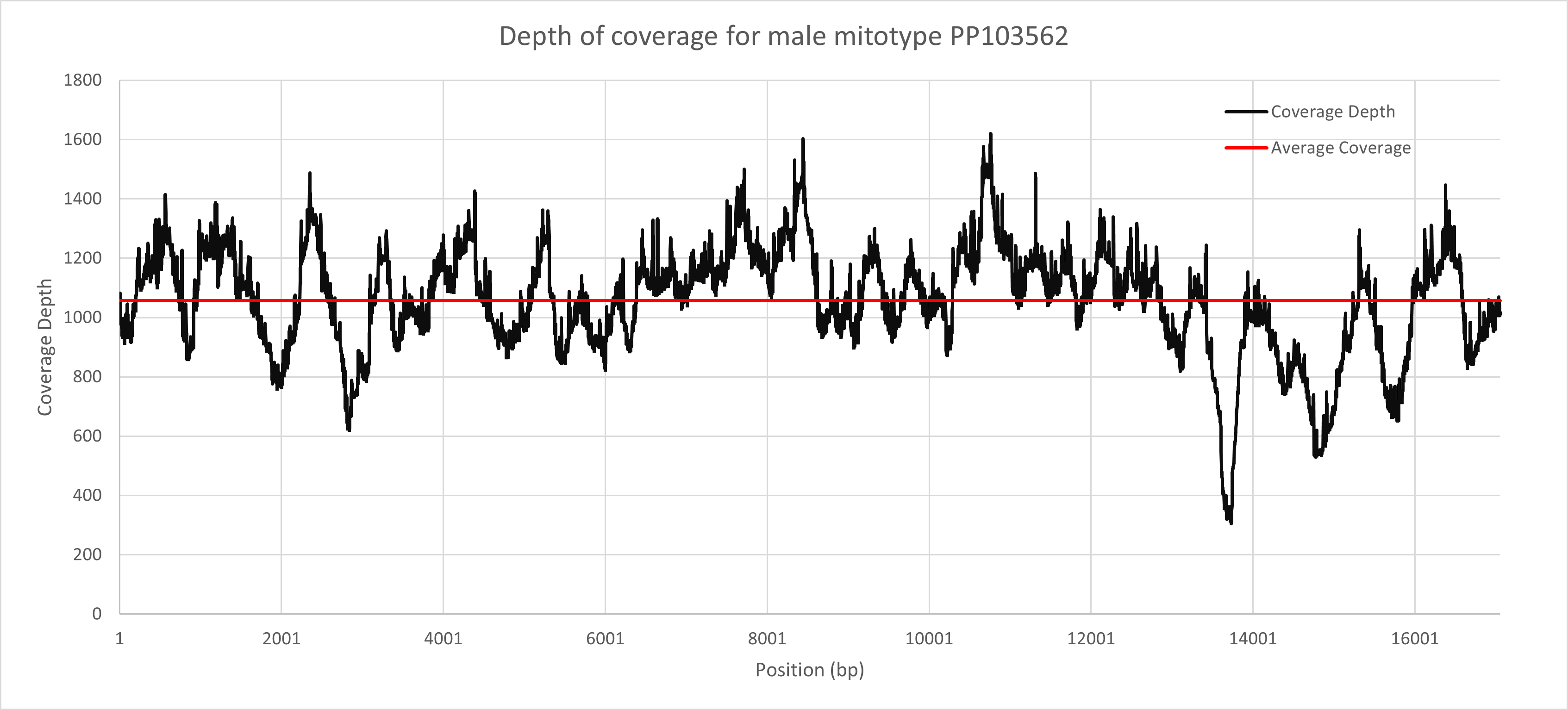


Figure S1. Read coverage plot of the male mitoype of *Ortmanniana ligamentina* from the Missouri individual (B065) (accession number PP103562).
